# Recent Progress of Exosome Isolation and Peptide Recognition-Guided Strategies for Exosome Research

**DOI:** 10.3389/fchem.2022.844124

**Published:** 2022-02-24

**Authors:** Kun Xu, Yulong Jin, Yongming Li, Yanyan Huang, Rui Zhao

**Affiliations:** ^1^ Beijing National Laboratory for Molecular Sciences, CAS Key Laboratory of Analytical Chemistry for Living Biosystems, CAS Research/Education Center for Excellence in Molecular Sciences, Institute of Chemistry, Chinese Academy of Sciences, Beijing, China; ^2^ School of Chemistry, University of Chinese Academy of Sciences, Beijing, China

**Keywords:** exosome, isolation, enrichment, peptide recognition, targeted therapy

## Abstract

Exosomes are membrane extracellular vesicles secreted by almost all kinds of cells, which are rich in proteins, lipids, and nucleic acids. As a medium of intercellular communication, exosomes play important roles in biological processes and are closely related to the occurrence, and development of many diseases. The isolation of exosomes and downstream analyses can provide important information to the accurate diagnosis and treatment of diseases. However, exosomes are various in a size range from 30 to 200 nm and exist in complex bio-systems, which provide significant challenges for the isolation and enrichment of exosomes. Different methods have been developed to isolate exosomes, such as the “gold-standard” ultracentrifugation, size-exclusion chromatography, and polymer precipitation. In order to improve the selectivity of isolation, affinity capture strategies based on molecular recognition are becoming attractive. In this review, we introduced the main strategies for exosome isolation and enrichment, and compared their strengths and limitations. Furthermore, combined with the excellent performance of targeted peptides, we summarized the application of peptide recognition in exosome isolation and engineering modification.

## Introduction

Exosomes are nanoscale extracellular vesicles secreted by almost all kinds of eukaryotic cells (Min et al., 2021) or bacteria (Vanaja et al., 2016; Tzipilevich et al., 2017). Unlike the outward blebbing of microvesicles, the biogenesis of exosomes involves a series of complex molecular regulation and substances exchange (Fei et al., 2021; Hao et al., 2021). It is generally believed that exosomes originate from the endosomes formed by the inward budding of plasma membrane (Jeppesen et al., 2019). Exosomes were once considered to be the waste products of cells in the original research. Nowadays, more and more studies have shown that exosomes play important roles in organisms, such as cell-to-cell communication (Ruhland et al., 2020; Schneider et al., 2021), immune response (Kalluri and LeBleu, 2020; Su et al., 2020), cell growth and differentiation (Yin et al., 2021; Zarnowski et al., 2021), as well as molecular transport (Zhu Q. et al., 2021). Crucially, the concentration and phenotype of exosomes have been proved to reflect the state of their parental cells and associate with the occurrence and development of various diseases, such as cancers (Jiang et al., 2021), infectious diseases (Ning et al., 2021; Xie et al., 2021), metabolic, and cardiovascular diseases (Kalluri and LeBleu, 2020). Therefore, developing exosome isolation methods with high efficiency and selectivity will definitely promote the deep understanding of the functions of exosome, as well as the accurate diagnosis and treatment of diseases.

Exosomes are widely existed in complex bio-systems, such as blood (Verweij et al., 2021), urine (Zheng et al., 2020), saliva (Wu et al., 2021), tears (Takeuchi et al., 2020), tissue fluid, and cerebrospinal fluid (Kalluri and LeBleu, 2020). Due to the heterogeneity of budding in the cell membrane, exosomes are not homogeneous vesicles but a series of complex subtypes with a size range from 30 to 200 nm and various functions respectively (Kalluri and Lebleu, 2020; Bordanaba-Florit et al., 2021). Besides, exosomes encapsulate various cargoes, including nucleic acids, proteins, lipids, and metabolites (Kowala et al., 2016; Mathieu et al., 2021). The contents of exosomes could be up- or down-regulated due to cell types or carcinogenesis (Cai et al., 2021). Such heterogeneity makes the isolation and enrichment of exosomes a challenging task.

A variety of exosome isolation protocols have been reported in the past few decades. Currently, ultracentrifugation has become the most accepted methods for exosome isolation (Théry et al., 2006; Yang et al., 2020), nevertheless it is often time-consuming and requires expensive instruments. Ultrafiltration (Liu et al., 2017), size-exclusion chromatography (Xu et al., 2016), and polymer precipitation (Pegtel and Gould, 2019) based on the physical characteristics (such as size and solubility) have been developed to achieve the isolation of exosomes. However, these methods usually capture the vesicles indiscriminately and cannot distinguish exosomes and lipoprotein particles efficiently. In order to improve the specificity, affinity-based capture strategies are emerging to isolate exosomes via the high binding abilities of specific exosomal markers to their corresponding ligands (Benjamin-Davalos et al., 2021). Because of the high specificity and affinity, antibodies are the most frequently used recognition tools (Ning et al., 2021; Park et al., 2021). Except for antibodies, peptides and aptamers have also been selected for exosome isolation (Gao et al., 2018; Chang et al., 2020; Zhu Lin et al., 2021). Through rational design and screening, specific protein-targeting peptides and aptamers can be obtained (Suwatthanarak et al., 2021a; Lin et al., 2021). Thus, highly selective capture and engineering modification of exosomes can be achieved effectively, which provide new strategies for exosome isolation and targeted therapy.

In this review, we introduced the main and novel strategies for exosome separation and enrichment, and discussed their strengths and limitations. Furthermore, we summarized the application of peptide recognition in exosome isolation and engineering modification.

## 2 Isolation and Enrichment Strategies of Exosomes

Obtaining exosomes with high yield, purity, and quality is the first step for the downstream analyses. However, exosomes are not single-component vesicles and have the complicated heterogeneity, which urgently demand the development of effective approaches for the isolation and enrichment of exosomes with high affinity and selectivity. After decades of efforts, several methods have been established, and successfully applied in exosome isolation from complex biosystems, such as “gold-standard” ultracentrifugation, size-exclusion chromatography, ultrafiltration, and polymer precipitation. Since these methods are based on the physical characteristics of exosomes, it is difficult to distinguish exosomes and interfering particles efficiently. To improve the selectivity of exosome enrichment, the exosomal markers provide new thoughts to develop affinity-based capture strategies based on the specific binding between such markers, and their corresponding ligands. Through this strategy, exosome subtypes expressing specific proteins can be efficiently isolated and enriched. In this section, we will introduce the conventional and new methods of exosome isolation and enrichment, and compare their advantages and limitations of each method.

### 2.1 Conventional Isolation and Enrichment Strategies

#### 2.1.1 Ultracentrifugation

**FIGURE 1:**
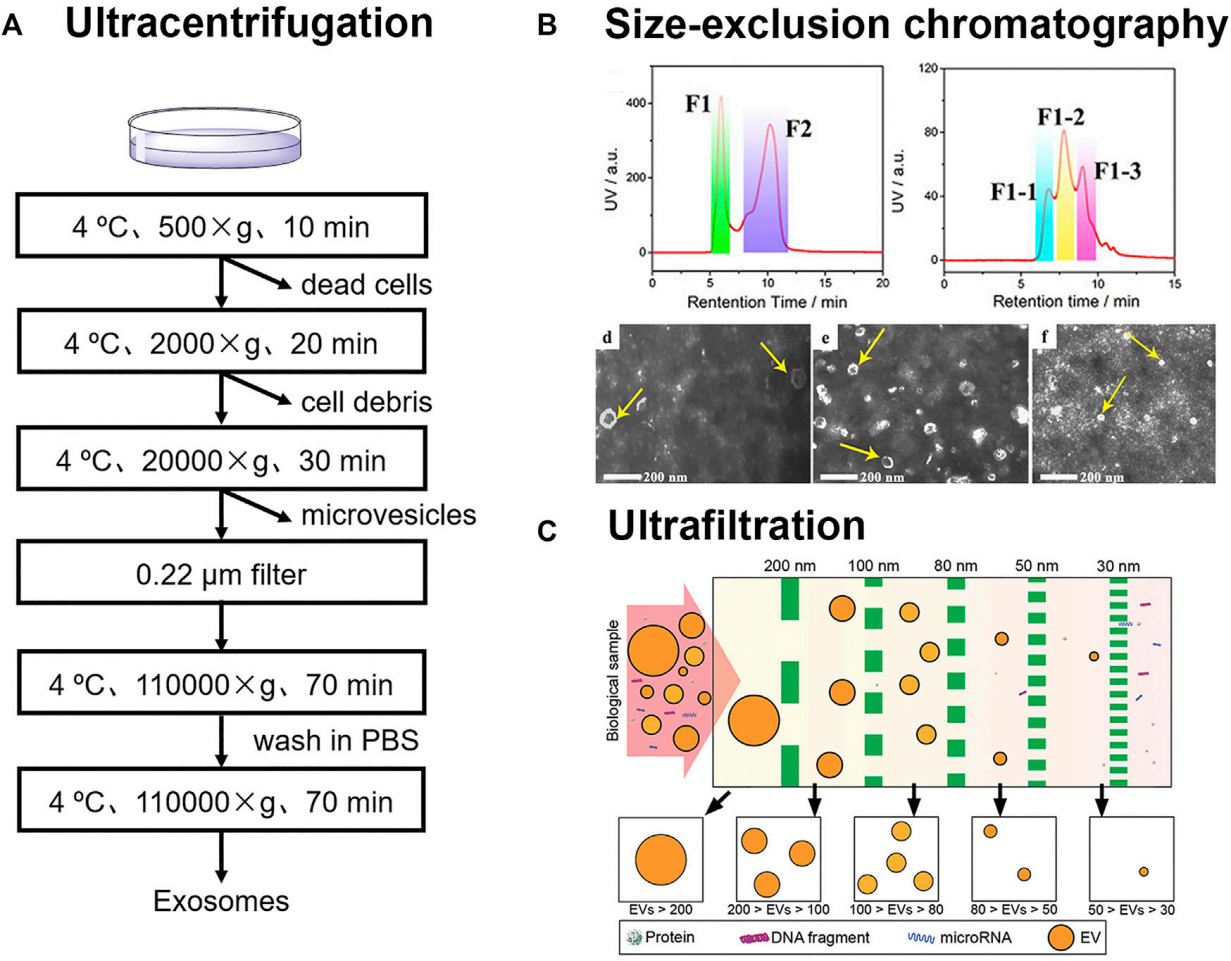
**(A)** Schematic for differential ultracentrifugation **(B)** Size-exclusion chromatography was employed for the isolation of three exosomes subpopulations from urines. Reprinted with permission from Zheng et al., 2020. Copyright 2020 American Chemical Society. **(C)** Exosomes were sorted into subtypes with different size distribution via ultrafiltration. Reprinted with permission from Liu et al., 2017. Copyright 2017 American Chemical Society.

Ultracentrifugation is the most widely accepted method for exosome separation, which usually includes differential ultracentrifugation, and gradient ultracentrifugation (Théry et al., 2006; Yang et al., 2020). This method can be employed to isolate exosomes from large scale of samples, such as more than 400 ml. However, the entire process usually takes more than 4 h and is very time-consuming (Shao et al., 2018). During the process of differential ultracentrifugation, cells, cell fragments, and large microvesicles in samples are removed successively under different centrifugal forces of 500, 2000, and 20,000 g (Figure 1A). Exosomes from cell culture medium are subsequently collected under ultracentrifugation conditions in excess of 1,10,000 g (Livshits et al., 2015). Li et al. successfully isolated exosomes from serum of breast cancer patients via differential ultracentrifugation (Li et al., 2018). Due to the high viscosity of serum, high centrifugal force of 1,50,000 g, and longer time of overnight were utilized.

During gradient ultracentrifugation, exosomes can be retained in the density equilibrium region (1.13–1.21 g/cm^3^) to isolate from other impurities (Melo et al., 2015). Paolini et al. isolated exosomes from serum of patients with multiple myeloma by differential ultracentrifugation, gradient ultracentrifugation, and one-step precipitation kits, and then assessed the presence of residual contaminants. The results showed that exosomes obtained by gradient ultracentrifugation were highly pure. In contrast, exosomes isolated by differential ultracentrifugation or one-step precipitation kits were contaminated by the residual matrix embedding the exosomes. The residual matrix prevented the fusion of the exosomes with plasma membrane, which interfered with the normal functioning of exosomes (Paolini et al., 2016). In general, exosomes can be obtained by gradient ultracentrifugation with higher purity, while differential ultracentrifugation has the advantage of separating large volume of samples at one time. However, it should be noted that high centrifugal force and repeated centrifugation may cause irreversible damage to vesicles.

#### 2.1.2 Size-Exclusion Chromatography

Size-exclusion chromatography (SEC) is a newly adopted method for the isolation of exosomes from complex samples (Suthar et al., 2020). In SEC columns, the diffusion paths of substances in the gels are different. The small vesicles and molecules in the samples have longer paths in the gels with a longer retention time, while the large vesicles have much shorter paths with a shorter retention time. Specifically, SEC method has good reproducibility and can also be used for quantitative detection of exosomes by fluorescence labeling (Xu et al., 2016).

The selection of gels has a significant effect on the isolation of exosomes. Xu et al. evaluated the separation efficiency of several commercially available SEC matrixes (Sepharose CL-2B, Sepharose CL-4B, Sephacryl S-100, and their combinations) for fluorescence-labeled exosomes from cell culture medium (Xu et al., 2016). The results showed that Sepharose CL-4B SEC column had the best separation efficiency, speed, and peak shape. Exosomes isolated from cell culture medium by this method showed a size range of 50–300 nm and the detection limit was calculated to be 2.9 × 10^7^ particles/mL. Guo et al. recently also investigated Sepharose CL-6B, CL-4B and CL-2B matrixes for exosome enrichments. Sepharose CL-6B was superior to CL-4B and CL-2B in the isolation of exosomes from protein serums (Guo et al., 2021). The exosomes with the size of 200 nm were collected. Since the pore sizes of CL-2B, CL-4B, and CL-6B are 75, 42, and 24 nm, respectively, exosomes with different sizes require different SEC separation materials.

SEC has also made great contributions in the research of exosome heterogeneity. Zheng et al. developed a two-dimensional SEC method for subtypes analysis of exosomes in urine (Zheng et al., 2020). Exosomes in urine were sorted into three subtypes (L-Exo, M-Exo, and S-Exo) in the 2^nd^ D-SEC (Figure 1B). Further, 144 glycoproteins and 44 phosphoproteins from L-Exo, 156 glycoproteins and 46 phosphoproteins from M-Exo, as well as 134 glycoproteins and 10 phosphoproteins from S-Exo were identified by liquid chromatography-tandem mass spectrometry. This confirmed that proteins in three subtypes of exosomes had different glycosylation and phosphorylation levels, which suggested that the corresponding exosomes may play different biological functions.

#### 2.1.3 Ultrafiltration

The size of vesicles is larger than that of biological molecules such as peptides, proteins, and nucleic acids. Vesicles can be intercepted by the membranes of different apertures. Poly (ether sulfone) (Heinemann et al., 2014), polycarbonate (Liu et al., 2017), and anodic aluminum oxide (Chen et al., 2021) can be used for the membrane filter. To address the need of sorting different-sized exosomes from the same samples, filters with different apertures can also be used in series (Figure 1C; Liu et al., 2017). However, the pore size of the membrane is usually narrow, and it is easy to clog when separating biological samples. It may also cause the deformation of vesicles under pressure.

Ultrafiltration has the advantages of fast, low cost, easy operation, and batch processing. Based on this, ultrafiltration is also feasible to achieve instrument automation. Chen et al. reported an efficient exosome isolation method via the ultrafast-isolation system (EXODUS) for varied biofluids (Chen et al., 2021). Double coupled harmonic oscillations were introduced into a dual-membrane filter configuration for the generation of transverse waves. The nanoporous membrane allowed small molecules and fluids to pass through, while exosomes remained inside the central chamber. EXODUS reduced the entire isolation time for 10 ml of urine to less than 10 min, while the isolation time of ultracentrifugation was more than 3 h. The exosomal transcriptome in 113 urine samples was profiled by the automation of EXODUS. According to the distribution analyses of RNA biotypes, mRNAs (33.1%), long noncoding RNAs (21.9%) and pseudogenes (21.7%) were identified as the most abundant biotypes.

#### 2.1.4 Polymer Precipitation

Polymer-based coprecipitation is also a common strategy in commercial exosome isolation kits, such as ExoQuickTM (System Biosciences, Unied States), ExoPrep (HansaBioMed, Estonia), and Total Exosome IsolationTM (Invitrogen, Unied States). Among various hydrophilic polymers, poly-ethylene glycol (PEG) is the widely used as the precipitation reagent. Hydrophilic PEG can interact with water molecules surrounding the exosomes, leading to a hydrophobic micro-environment (Yang et al., 2020). During this process, the solubility of exosomes decreases, and exosomes will precipitate under low-speed centrifugation. These commercial kits own high yields and easy adaptation to different researches. Meanwhile, this method avoids the utility of expensive ultracentrifugation and reduces the damage to vesicles. But it is worth noting that the process of polymer precipitation may also coprecipitate proteins, nucleic acids, and lipids (Paolini et al., 2016; Shao et al., 2018). Purity is an important factor that could cause interference during down-stream analysis. Therefore, it is very important to develop an exosome extraction kit with high purity and high yield. The mechanism, advantages, and limitations of the above-mentioned strategies for exosome isolation and enrichment have been summarized in Table 1.

**TABLE 1:** Comparison of strategies for exosome isolation and enrichment.

Strategy	Mechanism	Advantages	Limitations	Reference
Differential ultracentrifugation	density	a standard protocol, large sample volume	low yields and specificity, long time (more than 4 h), low purity, needs ultracentrifuge	Shao et al. (2018); Théry et al. (2006)
Gradient ultracentrifugation	density	a standard protocol, high purity	low yields, long time (more than 4 h), more complex operation steps, needs ultracentrifuge	Shao et al. (2018); Paolini et al. (2016)
Size-exclusion chromatography	size	fast and easy preparation, both small and large sample volume	low specificity, relatively high device cost	Zheng et al. (2020); Guo et al. (2021)
Ultrafiltration	size	fast and easy preparation, both small and large sample volume	low specificity, possible loss due to clogging	Liu et al. (2017); Chen et al. (2021)
Polymer precipitation	solubility	easy preparation, high yields, large sample volume	low specificity, long processing time (more than 12 h)	Yang et al. (2020)
Affinity capture	molecular recognition	high purity, suitable for small volume diagnosis	requires high-cost antibody, needs to select exosome markers	Chang et al. (2020); Yang et al. (2020)
Microfluidic technique	multiple principles, including affinity or size	portable and integrable, cost-efficient, fast preparation, high purity	low sample capacity, complex fabrication	Lin et al. (2020)

### 2.2 New Isolation and Enrichment Strategies

Exosomes are widely distributed in complex biological systems, where many interfering particles exist, such as lipoprotein particles, protein aggregates, and microvesicles with similarity in size and structure to exosomes. As shown in Table 1, the isolation methods based on the physical properties of exosomes are generally less specific. Therefore, how to effectively distinguish exosomes from interfering particles is a key problem to be solved. Meanwhile, biological samples are often precious and difficult to be obtained, which makes the isolation of exosomes from small-volume samples a great challenge. Therefore, affinity-based capture strategies, microfluidic chips, molecularly imprinted polymer, asymmetric-flow field-flow fractionation, and other technologies are gradually established.

#### 2.2.1 Affinity Capture with Biopolymers

Affinity capture strategy has been attractive in the isolation and enrichment of exosomes. Owing to the high affinity and excellent specificity, antibodies are the most frequently used recognition tools (Lin et al., 2021; Park et al., 2021). It is generally believed that certain transmembrane proteins (such as CD9, CD63, and CD81) are highly expressed on the surface of exosomes. Ning et al. described an assay approach where exosomes were directly captured from COVID-19 patient plasma through the interaction of an antibody with the exosomal surface protein CD81 (Ning et al., 2021). Then liposomes containing reagents for reverse transcriptase were introduced to realize the ultrasensitive detection of SARS-CoV-2 RNA. SARS-CoV-2-positive exosomes were detected early at 1 day post-infection in a non-human primate model. Meanwhile, this approach offered a powerful tool for the diagnosis of patients with COVID-19. In addition, tumor-derived exosomes have also been identified to contain corresponding tumor biomarkers (Min et al., 2021), such as EpCAM, PD-L1, and EGFR. Li et al. established a strategy for the disease diagnosis based on microbead-assisted flow cytometry. Exosomes were enriched by aldehyde beads and flow cytometry was then performed to detect the disease markers by using corresponding antibodies. Using these strategies, they succeeded in achieving a highly sensitive diagnosis of breast cancer (Li et al., 2018) and invasive nonfunctional pituitary adenomas (Wang et al., 2019).

**FIGURE 2:**
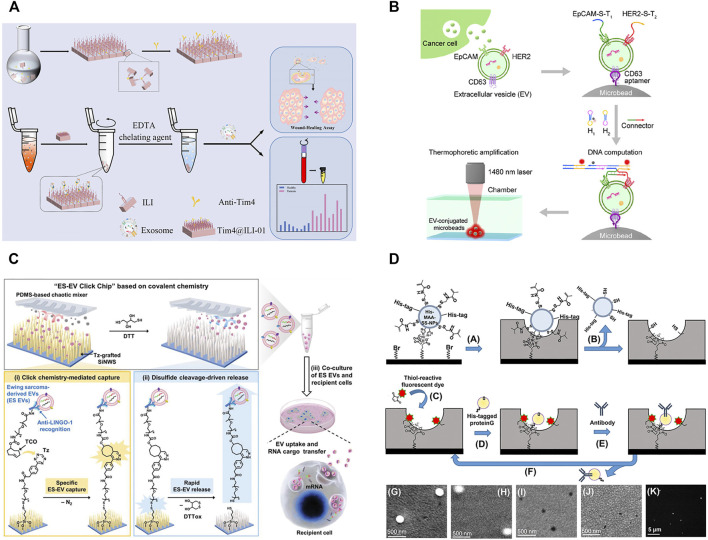
**(A)** Schematic for synthesis of Tim4@ILI-01 immunoaffinity flake and exosomes enrichment and downstream analysis. Reprinted with permission from Zhang et al., 2021a. Copyright 2021 American Chemical Society. **(B)** Schematic of aptamer-based isolation of extracellular vesicles. Reprinted with permission from Li et al., 2021. Copyright 2021 American Chemical Society. **(C)** Schematic for nanowire-embedded microchip’s working mechanism and purification of exosomes. Reprinted with permission from Dong et al., 2020. **(D)** Molecular imprinting-based nanocavities for sensing intact exosomes. Reprinted with permission from Takeuchi et al., 2020. Copyright 2020 American Chemical Society.

Although antibodies reveal the high affinity and excellent specificity in exosome isolation, antibodies also have some limitations, such as the complex preparation process, expensive costs, and low stability (Chang et al., 2020). Compared with antibodies, aptamers and peptides show the advantages of easy chemical synthesis, flexible modification, and high stability, which have expanded their applications in exosome isolation and enrichment (Zhu L. et al., 2021; Chang et al., 2020). Li et al. modified the microbeads with CD63-aptamers and developed a new thermophoretic aptasensor for the separation and detection of exosomes (Figure 2B; Li et al., 2021). In this process, exosomes in the samples were first captured by CD63-aptamers to the surface of microbeads. Then, infrared laser was used for local heating to lead to the directional enrichment of microbeads. Employing this platform, breast cancer patients and healthy donors could be discriminated with a high accuracy of 97%. This strategy was also successfully applied in therapeutic response in metastatic breast cancer (Tian et al., 2021) and the classification of prostate cancer (Liu Chao et al., 2019). Chang et al. reported an CD63-aptamer-based magnetic graphene composites to achieve the convenient capture and efficient enrichment of exosomes from cell culture medium (Chang et al., 2020). The prepared composites were then applied to profile the metabolite composition of MCF-7- and MCF-10A-derived exosomes, and 119 metabolites were identified in total. Compared with exosomes from MCF-10A, 43, and 42 metabolites were upregulated and downregulated in exosomes from MCF-7.

During the biogenesis of vesicles, phosphatidylserine is regulated by the flippase and distributed in the outer membrane of exosomes (Shao et al., 2018). Therefore, the highly expressed phosphatidylserine can also be used as a target for the isolation of exosomes. TiO_2_ is often modified on the surface of magnetic beads to separate exosomes via the non-covalent bond of Ti-PO_3_-Ti (Pang et al., 2020). In addition, phosphatidylserine can also specifically bind to Tim4 protein and is identified to be Ca^2+^-dependent. Magnetic beads immobilized with Tim4 can capture exosomes quickly, and intact exosomes can be easily eluted by chelating agent (Xu et al., 2018). Zhang et al. developed a novel Tim4@ILI-01 immunoaffinity flake material for the enrichment of exosomes from serum (Figure 2A; Zhang et al., 2021b). The gene analysis of eluted exosomes showed that the level of the expressed CD44 gene was significantly high in lung adenocarcinoma patients. The captured exosomes significantly induced more migration than the uneducated cells via a wound-healing assay, and the expression of epithelial-mesenchymal transition-related proteins changed significantly during cell migration. Since phosphatidylserine is not unique to exosomes and is also found in other vesicles, such as microvesicles, the specificity of this method remains to be assessed.

#### 2.2.2 Molecular Imprinting Technique

As a kind of artificial antibodies, molecularly imprinted polymers (MIPs) have the unique advantages of high specificity, good chemical stability, tailor-made versatility (BelBruno, 2018). Shape, size, and spatial matching play the indispensable roles in the recognition process of MIPs with exosomes. Yang et al. reported a surface imprinting technology for antibody-free magnetic isolation of small extracellular vesicles (sEVs) (Yang et al., 2021). sEVs bound to the magnetic beads were easily removed by mild ultrasonic treatment, and vesicles could be isolated by the imprinted holes. The MIPs presented a higher capture yield in 20 min, which was 3-fold enrichment of sEVs compared with ultracentrifugation (more than 4 h). CD24 and EpCAM on sEVs were highly overexpressed in phenotype analyses, providing an effective predictive tool for real-time noninvasive monitoring of tumor development in mice. Mori et al. reported a kind of molecular imprinting-based antibody-conjugated nanocavities (Mori et al., 2019). Exosomes in tears were captured to the surface of MIPs by the double recognition of antibody and nano holes. The MIPs were successfully employed to discriminate normal exosomes and prostate cancer patients’ exosomes in tear drops. However, when exosomes were used as the templets for exosome imprinting, it was usually difficult to synthesize MIPs with uniform pore sizes. Aslo, during the polymerization, the integrity and surface properties of exosomes might be destroyed. To overcome these drawbacks, silica nanoparticles have been used to simulate exosomes, aiming to create MIPs with uniform pore sizes (Figure 2D; Takeuchi et al., 2020). The apparent dissociation constant of MIPs to exosomes was calculated to be 2.4 × 10^–16^ mol/L, which was almost 1000-fold lower than that of commercial immunoassays. Exosomes from tears were successfully captured and used for the noninvasive diagnosis of breast cancer. Even though, the preparation routes of MIPs are still relatively complex and required multi-step chemical modification (BelBruno, 2018). Meanwhile, the reproducibility and affinity of MIPs need to be improved.

#### 2.2.3 Microfluidic Technique

Conventional exosome separation and purification methods often require a large volume of samples, complicated operation, or long time consuming. Microfluidics as a new technology can manipulate tiny fluids (ranging from a few microlitre to hundreds of microlitres) in microtubes. Microfluidic chip owns unique advantages of fast separation speed, high throughput, and less required samples, which is very suitable for the separation of exosomes from few precious biological samples (Lin et al., 2020; Hassanpour Tamrin et al., 2021).

Antibodies, aptamers, or peptides are often modified in microfluidic channels to increase the specificity of exosome isolation (Xu et al., 2018; Suwatthanarak et al., 2021b; Yu et al., 2021). Furthermore, in order to enhance the opportunities of collisions between exosomes and antibodies, aptamers, or peptides, a variety of micropipe shapes have been rationally designed for exosome enrichment, and such as trapping microchannel (Tayebi et al., 2020), Y-shaped micropillars (Xu et al., 2018), and nanowires (Sun Na et al., 2020). Their combination will help to improve the selectivity and efficiency of exosome isolation. Yu et al. reported a highly integrated exosome separation and detection chip (ExoSD) which was modified with anti-CD63 antibodies (Yu et al., 2021). Exosomes were efficiently isolated from cell culture supernatant and clinical serums of gastric cancer patients (stages I and II). To isolate Ewing sarcoma-derived exosomes, Dong et al. developed a specific purification system by a silicon nanowire-embedded microchip conjugated with antibodies (Figure 2C; Dong et al., 2020). The purified exosomes could be released intactly by disulfide cleavage and internalized once more by recipient cells, transferring their RNA cargoes *in vivo* to exhibit their potential roles in intercellular communication.

In addition, the isolation and analysis of single cell-derived exosomes is still a scientific problem. Zhu et al. designed a microwell array chips to trap a single cell, and antibody-coated glass slide could capture exosomes secreted from the single cell (Zhu F. et al., 2021). The number of exosomes secreted from every single cell was quantified by combining gold nanoparticle-enhanced silver staining, which reflecting the ability of individual cells to secrete exosomes. A very few cells (2–3%) in the cell line secreted exosomes 60 to 80 times faster than the other cells. If these cells were excluded, the total number of exosomes secreted would reduce by 2/3. This strategy would also provide a powerful tool for the research of other precious and rare cells with minimal consumption, such as circulating tumor cells.

#### 2.2.4 Asymmetric-flow field-flow Fractionation

Asymmetric-flow field-flow fractionation (AF4) is a powerful fractionating technique with great flexibility to separate samples in a large size range, such as proteins, virus, liposomes, and various polymers (Kim et al., 2020). Compared with ultracentrifugation and ultrafiltration, AF4 could obtain intact extracellular vesicles easily without high centrifugal force and pressure (Zhang and Lyden, 2019). By employing AF4, Zhang et al. identified two exosome subtypes (large exosome vesicles, 90–120 nm and small exosome vesicles, 60–80 nm) and discovered an abundance of non-membranous nanoparticles termed “exomeres” (∼35 nm) (Zhang et al., 2018). Compared with exosomes, exomeres had unique N-glycosylation, proteins, lipids, DNA, and RNA profiles. These subsets were proved to diverse organ biodistribution, playing distinct biological functions. However, in the current study, AF4 for exosome isolation are usually lengthy duration and limited by the expensive equipment.

#### 2.2.5 Liposome Fusion Technique

Liposomes and exosomes have the similar membrane structures, and the fluidity of the bilayer membrane allows the fusion of liposomes and exosomes. This principle has also been applied to the isolation and content analysis of exosomes (Ning et al., 2021). Liu et al. designed an antibody-modified lipid patch microarray for rapid capture of cancer extracellular vesicles (Liu Hui-Yu et al., 2021). The binding exosomes successfully fused with the lipid layer to trap the contents to the surface of the microarray. The RNA cargo trapped in the lipid patches offered a high potential for the downstream analysis.

## 3 Peptide Recognition-Guided New Strategies for Exosome Research

Molecular recognition involves almost every link of life process and plays an important role in the growth, development, metabolism, and aging process of life. As a classical molecular recognition tool, antibody has been widely used in the recognition and research of target molecules. Meanwhile, peptides are one of the most crucial biomolecules in life, which are formed by dehydration of various amino acids. Compared with biological macromolecules, peptides have their unique advantages, such as high stability, diverse properties, and easy preparation (He et al., 2017). The rapid development of solid-phase synthesis strategy offers great convenience for the automatic batch synthesis and site-specific chemical modification of peptides (Pedersen et al., 2012).

In recent years, the applications of peptides towards exosome research received more and more concentration. Exosome-anchoring peptides were obtained by the rational design and screening from peptide libraries (Liu Xiangwei et al., 2019). The sequences and targets of exosome-targeting peptides have been shown in Table 2. Exosome-targeting peptides with high binding affinity and selectivity were successfully applied for the efficient separation and enrichment of exosomes. Furthermore, cargo molecules coupled with peptides can be easily modified to exosomes via a non-covalent way, which play a huge role in drug delivery, and treatment of diseases.

**TABLE 2:** The sequences and targets of exosome-targeting peptides.

Peptide	Sequence	Target	References
CP05	CRHSQMTVTSRL	CD63	Gao et al. (2018)
P238	RSHRLRLH	CD9	Suwatthanarak et al. (2021a); Suwatthanarak et al. (2021b)
Vn96	PSQGKGRLSLSRFSWGALTLGEFLKL	Heat shock protein 70	Ghosh et al. (2014); Bijnsdorp et al. (2017); Bathini et al. (2021)
BP	RPPGFSPFR	Exosome membranes	Saludes et al. (2013); Gori et al. (2020)
PS-specific peptide	FNFRLKAGAKIRFGRGC	Phosphatidylserine	Liu X. et al. (2021)
EGFR-specific peptide	FALGEA	Epidermal growth factor receptor	Sun Z. et al. (2020)

### 3.1 Peptide Recognition-Based Exosome Isolation and Enrichment

To obtain a new exosome-targeting peptide, the highly expressed molecules in exosomes can be selected as the targets, such as transmembrane proteins (CD9, CD63, or CD81), tumor markers, and or phosphatidylserine on biofilms. Gao et al. selected the second extracellular loop of CD63 as a target and obtained a high affinity exosome-targeting peptide CP05 by phage display technology (Gao et al., 2018). CP05 can bind to the surface of exosomes efficiently (binding efficiency up to 88.7%) without changing the original properties of exosomes. When CP05 was fixed on dynabeads, 108.98 ± 7.82 μg of exosomes could be captured efficiently from per milliliter of serum. For another exosomal marker protein CD9, Suwatthanarak et al. designed and synthesized a candidate peptide library in a microporous array, in which the peptide sequences were derived from the CD9 partner protein EWI-2 (Suwatthanarak et al., 2021a). A CD9-targeting peptide P238 was screened out, revealing a dissociation constant of 4.66 × 10^–7^ mol/L. In order to further explore the binding mechanism of P238 to CD9, a site-by-site alanine substitution of P238 was performed. The results showed that the binding ability decreased significantly after the substitution of arginine, histidine, and serine. This also suggested that electrostatics and polarity played important roles in the binding of P238 to CD9 (Suwatthanarak et al., 2021b).

Besides, tumor-targeting peptides can accurately locate the tumor-derived exosomes from numerous exosomes, and provide a powerful tool for the early diagnosis of tumors. Sun et al. designed an electrochemical sensor, combining EPGR-targeted peptides with Zr-MOFs for the quantification of exosomes from glioblastoma (Sun Zhaowei et al., 2020). The detection range was from 9.5 × 10^3^–1.9 × 10^7^ particles/μl, and the detection limit was 7.83 × 10^3^ particles/μl. The signals of exosomes from Glioblastoma patients were significantly higher than healthy samples, being consistent with the higher expression of EGFR in Glioblastoma-derived exosomes than normal cells.

Heat shock protein (HSP) is highly expressed in exosomes. When HSP-targeting peptide Vn96 was bind to exosomes, the hydrophilicity of exosomes might be changed, and reducing the solubility of exosomes (Ghosh et al., 2014). Inspired by this principle, Bijnsdorp et al. designed a new exosome isolation kit to obtain exosomes with low-speed centrifugation (Bijnsdorp et al., 2017). By the excellent binding ability of Vn96, Bathini et al. presented an immunoaffinity chip for the capture of exosomes from the MCF-7 cell culture medium (Bathini et al., 2021). The isolation efficiency obtained was about 90% when 200 μl MCF-7 cell culture medium was added.

In addition, curvature-targeting peptide (Saludes et al., 2013) and phosphatidylserine-targeting peptide (Liu Xuejiao et al., 2021) have also been reported for exosome isolation and enrichment. Curvature-targeting peptide (BP) rich in positive-charged amino acids (e.g., arginine) can bind to the negative-charged exosome membranes via the electrostatic interaction. Meanwhile, the secondary helical structure can induce the peptides to be embedded in the defect of membranes, thus increasing the anchoring ability of peptides. Gori et al. designed a microarray platform modified with curvature-targeting peptide to isolate exosomes from serum without pretreatment (Gori et al., 2020). Fluorescence imaging results showed that the captured exosomes ranged a particle size of 50–120 nm and were rich in CD63, CD81, and CD9.

### 3.2 Peptide-Engineering Exosomes for Targeted Therapy

Since the first clinical trial in the treatment of metastatic melanoma patients (Escudier et al., 2005), exosomes have attracted more and more attention in the field of drug delivery and disease treatment (Meng et al., 2021; Xie et al., 2021). As a platform for drug delivery and disease treatment, exosomes have several significant advantages. Firstly, homing effect is the unique advantage of exosomes compared with liposomes (Zhang et al., 2017). Tumor-derived exosomes have natural targeting ability to tumor cells. Secondly, the membrane of exosomes protects the contents from release in the circulatory system (Zhu et al., 2020). After exosomes are fused with the target cell membrane, drugs loaded by exosomes can be released efficiently. Thirdly, exosomes secreted by cells are low immunogenic (Kalluri and Lebleu, 2020), such as plant- or milk-derived exosomes.

For drug loading, electroporation is a widely used method, however it can damage the membrane structure of exosomes (Gudbergsson et al., 2019). Different from electroporation, using exosome-targeting peptide as a bridge, the peptide-drug conjugates can be anchored to the surface of exosomes via the non-covalent interaction between the peptides and exosomes in a mild condition, which do not affect the original physiological activity of exosomes.

**FIGURE 3:**
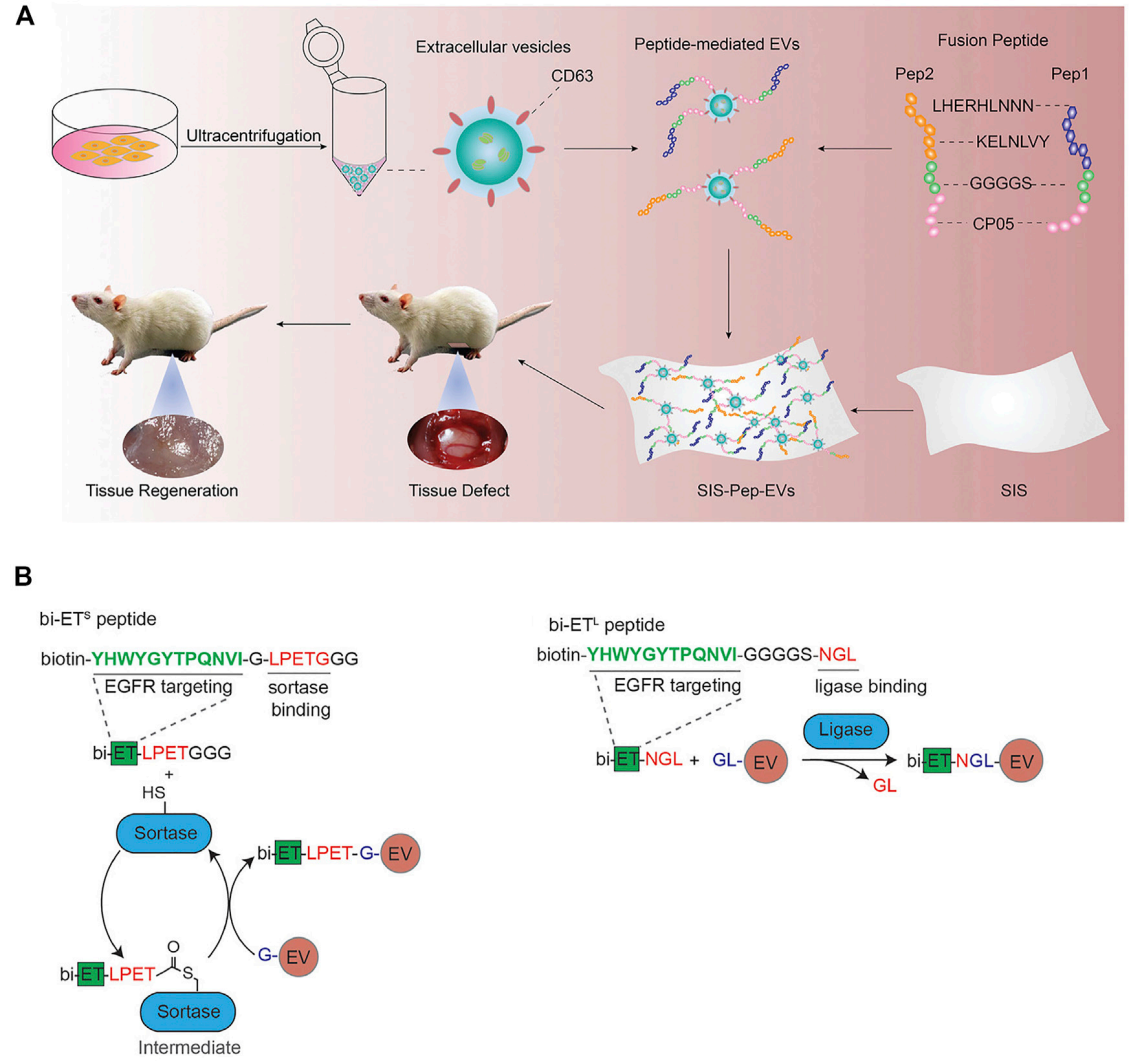
**(A)** Schematic diagram of the membrane modified by fusion peptide-mediated exosomes. Reprinted with permission from Zhang et al., 2021b. **(B)** Protein ligating enzymes mediate a covalent conjugation of exosomes with peptides. Reprinted with permission from Pham et al., 2021.

As described in the previous section, CP05 is an excellent exosome-targeting peptide that can anchor to the exosomal surface protein CD63. Gao et al. constructed a ternary complex with an oligonucleotide drug PMO, a muscle-targeting peptide M12 and an exosome-targeting peptide CP05. By the targeted recognition of CP05, exosomes were successfully loaded PMO and M12. The muscle-targeting peptide functionalized exosomes can accurately deliver oligonucleotide drugs to muscle tissue for the treatment of duchenne muscular dystrophy (Gao et al., 2018). Besides, CP05 was also successfully applied in the treatment of other several diseases, including tumor immunotherapy (Zuo et al., 2020), proliferative retinopathy (Dong et al., 2021), and traumatic optic neuropathy (Wang et al., 2021). CP05 can also work synergistically with other targeted peptides. For example, two collagen-binding peptides (LHERHLNNN and KELNLVY) were combined with CP05 to promote the engineering modification of exosomes derived from umbilical cord mesenchymal stem cells (Figure 3A; Zhang et al., 2021a). The peptide-exosome conjugates could improve tissue regeneration and were successfully used to the treatment of tissue injury.

In addition to these engineering approaches, it has also been reported that peptide can be inserted into exosomal surface proteins by transfection or ligase (Gudbergsson et al., 2019). Pham et al. used protein ligase to introduce EGFR-targeting peptides and HER2-targeting peptides to the surface of exosomes (Figure 3B; Pham et al., 2021), which could improve the targeting ability of exosomes loaded with paclitaxel and achieve satisfied therapeutic effects.

## 4 Conclusion

With an extensive research interest in exosomes in recent years, a variety of methods based on different principles have been developed by researchers for the isolation and enrichment of exosomes from biofluids. Great progress has been achieved regarding the isolation performance, including yield, purity, quality, and efficiency. However, as summarized above, every method has its inherent merits and shortcomings based on these evaluation index. Therefore, it is wise and highly recommended to select the right isolation method or method combination that well suits the aim of downstream research on exosomes. Despite of the progress, there is much space for growth in this area. The development of novel materials, isolation methods that hold a better performance, as well as integrating them with online analytical instruments will be promising to achieve a more accurate and efficient isolation and analysis of exosomes in the years to come. Furthermore, the screening of exosome-targeting peptides as the affinity materials and developing new methods for the construction of peptide-exosome complex will provide useful tools for the peptide-recognition guided exosome research and applications.
